# TREC/KREC Levels in Young COVID-19 Patients

**DOI:** 10.3390/diagnostics11081486

**Published:** 2021-08-16

**Authors:** Maryam B. Khadzhieva, Ekaterina V. Kalinina, Sergey S. Larin, Daria A. Sviridova, Alesya S. Gracheva, Julia V. Chursinova, Vadim A. Stepanov, Ivan V. Redkin, Lyudmila S. Avdeikina, Alexander G. Rumyantsev, Artem N. Kuzovlev, Lyubov E. Salnikova

**Affiliations:** 1 Federal Research and Clinical Center of Intensive Care Medicine and Rehabilitology, 107031 Moscow, Russia; palesa@yandex.ru (A.S.G.); redkin70@mail.ru (I.V.R.); artem_kuzovlev@fnkcrr.ru (A.N.K.); salnikovalyubov@gmail.com (L.E.S.); 2 Dmitry Rogachev National Research Center of Pediatric Hematology, Oncology and Immunology, 117997 Moscow, Russia; ek.v.chernyshova@gmail.com (E.V.K.); sergei_larin@mail.ru (S.S.L.); alexrum47@mail.ru (A.G.R.); 3 Vavilov Institute of General Genetics, Russian Academy of Sciences, 119991 Moscow, Russia; daria_sv11@mail.ru; 4 M.F. Vladimirsky Moscow Regional Research and Clinical Institute, 129110 Moscow, Russia; yu.chursinova@monikiweb.ru (J.V.C.); vedmak_@rambler.ru (V.A.S.); 5 Moscow Clinical Center for Infectious Diseases “Voronovskoe”, 142160 Moscow, Russia; saenko.doc@gmail.com

**Keywords:** COVID-19, acute respiratory distress syndrome, TREC/KREC, neutrophil-to-lymphocyte ratio

## Abstract

COVID-19 patients with acute respiratory distress syndrome (ARDS) have an immune imbalance when systemic inflammation and dysfunction of circulating T and B cells lead to a more severe disease. Using TREC/KREC analysis, we studied the level of mature naive T and B cells in peripheral blood of COVID-19 patients and its relationship with clinical and laboratory data. TREC/KREC analysis was performed by multiplex real-time quantitative PCR on a sample of 36 patients aged 45 years or younger. The reduced TREC/KREC level was observed in ARDS patients compared with non-ARDS patients, and similar results were found for the deceased patients. During days 6 to 20 of hospitalization, a higher neutrophil-to-lymphocyte ratio (NLR) was detected in ARDS patients compared with non-ARDS patients. TREC/KREC negatively correlated with NLR; the highest correlation was recorded for TREC per 100,000 cells with the coefficient of determination R^2^ = 0.527. Thus, TREC/KREC analysis is a potential prognostic marker for assessing the severity and outcome in COVID-19.

## 1. Introduction

The pandemic of the Coronavirus disease 2019 (COVID-19) caused by SARS-CoV-2 (family *Coronaviridae*, genus *Betacoronavirus*) continues to be a major challenge worldwide. Most patients with COVID-19 have a mild disease; however, about 20% of those infected need to be hospitalized because of pneumonia [1]. The severe COVID-19 most often manifests as acute respiratory distress syndrome (ARDS) with significant abnormalities, including air–blood barrier damage, systemic inflammation, extensive lung injury, immune system dysfunction, and secondary infections [2]. ARDS is reported in over 70% of COVID-19 patients hospitalized in the intensive care unit [3,4]. Severe course and unfavorable outcomes in hospitalized COVID-19 patients can be associated with lymphopenia in combination with neutrophilia [5,6,7,8]. Restoration of lymphocyte count may be important for a patient’s recovery not only because of the impaired adaptive antiviral response in lymphopenia, but also due to an increased risk of severe hyperinflammatory response in COVID-19 [9].

The maturation of functional human T and B cells is accompanied by recombination and rearrangements in the genes encoding T and B cell receptors and the formation of extrachromosomal circular DNA, which is not capable of replication including TREC (T cell receptor excision circle) forming during maturation of naive T cells in the thymus and KREC (kappa-deletion recombination excision circle) developing during maturation of naive B cells in bone marrow. Determination of TREC/KREC ratio in peripheral blood is mainly used for screening of primary immunodeficiencies associated with impaired maturation of T and B cells [10,11], and to assess immune system functioning after hematopoietic stem cell transplantation [12], to determine response to antiretroviral therapy of HIV infection [13], as well as to study and evaluate the effectiveness of therapy for lymphoproliferative diseases [14].

We examined the level of mature naive T and B cells in peripheral blood using TREC/KREC analysis in relation to the development of ARDS and death in COVID-19, with consideration of clinical and laboratory data.

## 2. Materials and Methods

### 2.1. Patients

The study included 36 patients diagnosed with COVID-19, who were hospitalized in M.F. Vladimirsky Moscow Regional Research and Clinical Institute and Moscow Clinical Center for Infectious Diseases “Voronovskoe” from 27 April to 28 November 2020. The diagnosis was confirmed by laboratory testing in accordance with the provisional guidelines on prevention, diagnosis, and treatment of the novel coronavirus infection (COVID-19) of the Russian Ministry of Health (Version 11). The Ethics Committee of the Federal Research and Clinical Center of Intensive Care Medicine and Rehabilitology approved the study; all included patients signed an informed consent. The presence of ARDS and the determination of its severity were carried out in accordance with the Berlin Definition [15].

Patients of both sexes aged 18 to 45 years, who signed the informed consent for study participation, without severe medical, immunological, and surgical comorbidities and/or complications, as well as pregnancy throughout the study were enrolled in the study. Exclusion criteria were terminal incurable diseases; primary and/or secondary immunodeficiency; pregnancy; and refusal to participate in the study.

### 2.2. DNA Extraction and Quantitative PCR Assay

Venous blood was collected in IMPROVACUTER^®^ EDTA Tubes (Guangzhou Improve Medical Instruments Co., Ltd., Guangzhou, China) and stored at −20 °C. DNA was isolated from 200 μL of venous blood by isopropanol precipitation. Primers and probes were designed to specifically amplify δREC-ψJα T cell receptor, kappa-deleting element, and human albumin (*ALB*) as reference gene. TREC, KREC, and albumin levels were simultaneously determined by multiplex real-time quantitative PCR (RQ-PCR) in 25 μL of reaction mixture containing 200 ng DNA. PCR protocol included 7 min at 95 °C followed by 45 cycles of 30 s at 93 °C and 1 min at 59 °C (CFX96, Bio-Rad, USA). The sequence of primers and probes is shown in Supplementary Table S1.

Standard curves for accurate quantification of TREC, KREC, and albumin were obtained by constructing a calibration curve from serially diluted genetic constructs containing the TREC/KREC junction region and the corresponding albumin gene region. TREC and KREC copies were calculated and expressed as copies per 100,000 nucleated cells and per WBC (white blood cell) and lymphocyte counts in 1 μL of blood as follows:

$$\mathrm{TREC}\;\mathrm{or}\;\mathrm{KREC}/100,000\;\mathrm{cells}=\frac{\mathrm{Copy}\;\mathrm{number}\;\mathrm{of}\;\mathrm{TREC}\;\mathrm{or}\;\mathrm{KREC}}{\mathrm{Copy}\;\mathrm{number}\;\mathrm{of}\;\frac{\mathrm{ALB}}{2}}\times 100,000$$

TREC or KREC/WBC(or lymph) in 1 µL of blood=(TREC or KREC copies)×WBC or lymph in 1 μL

### 2.3. Statistical Analysis

Statistical analysis and data visualization were performed using IBM SPSS Statistics 25.0 (IBM, New York, NY, USA) and R software package (RStudio 1.4, https://www.rstudio.com, accessed on 19 May 2021). Data for quantitative nonparametric variables are presented as medians and 10th and 90th percentiles, and as values and percentage for categorical data. Comparative intergroup analysis was performed using the nonparametric Mann–Whitney U-criterion, and correlation analysis was performed by calculating the Spearman correlation coefficient. Dichotomous data were analyzed using Fisher’s exact two-sided F-criterion. The significance level at which the null hypothesis of no difference between the study groups was rejected was set at 0.05. Correction for multiple comparisons was performed using the Benjamini–Hochberg method (FDR, False Discovery Rate).

## 3. Results

The total sample size was 36 patients; 10 (27.78%) patients had ARDS, and 4 (11.11%) of them did not survive. The median age (10th and 90th percentiles) was 38.50 (31.00 to 44.00) years. More than half of the study participants were males (58.3%). The most frequently reported findings on admission were fatigue (86.11%), cough (66.67%), fever (61.11%), and dyspnea (36.11%). The main patient characteristics are shown in Table 1.

Comparative analysis of clinical and laboratory data and TREC/KREC levels was performed between patients with ARDS (*n* = 10) and without it (*n* = 26). No differences were found between these groups with respect to age, sex, day of illness at the time of analysis, WBC, RBC (red blood cells), and platelet counts. Patients with ARDS differed from the non-ARDS group ones in reduced lymphocyte count (*p* = 0.014), increased neutrophil count (*p* = 0.049), and neutrophil-to-lymphocyte ratio (NLR) (*p* = 0.002). When adjusted for multiple comparisons, significant differences were reported only for NLR (FDR adjusted *p*-value = 0.022) (Supplementary Table S2).

**Figure 1:**
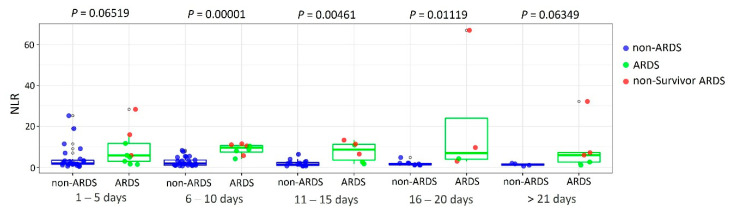
Comparison of NLR between ARDS and non-ARDS COVID-19 patients.

For NLR, a comparative analysis was performed between patients with and without ARDS from the time of hospitalization to discharge or patient death in five-day increments. Differences were found at 6 to 10 (*p* = 0.00001; FDR adjusted *p*-value = 0.00001), 11 to 15 (*p* = 0.00461; FDR adjusted *p*-value = 0.01153), and 16 to 20 (*p* = 0.01119; FDR adjusted *p*-value = 0.01865) treatment days (Mann–Whitney U test) (Figure 1).

Analysis of TREC/KREC levels both per 100,000 cells and per leukocyte and lymphocyte counts per 1 µL of blood revealed significant differences: TREC/KREC values were lower in the group of ARDS patients; these differences persisted after adjustment for multiple comparisons (Table 2). The TREC/KREC levels were also lower in non-survivors than in survivors (Supplementary Table S3).

**Figure 2:**
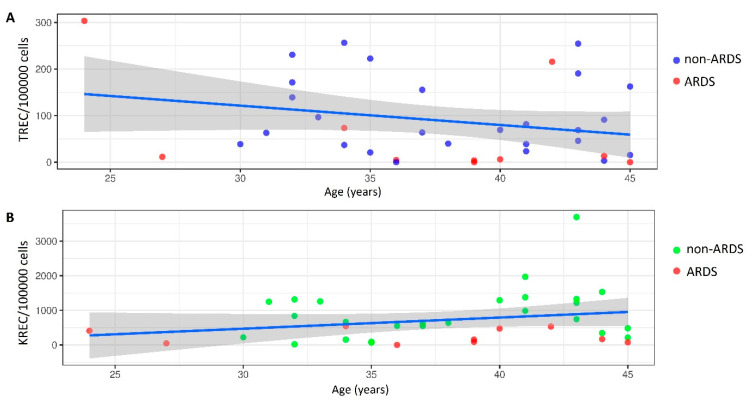
Linear regression analysis of age and TREC/KREC in ARDS and non-ARDS patients: (**A**) TREC; (**B**) KREC.

Linear regression analysis revealed no correlation of TREC/KREC level with patient age, length of stay, and day of biomaterial collection from the onset of COVID-19 symptoms. The age-specific distribution of TREC/KREC per 100,000 cells is shown in Figure 2 (TREC: R^2^ = 0.065, *p* = 0.134; KREC: R^2^ = 0.059, *p* = 0.155).

Correlation analysis of TREC/KREC values revealed a significant inverse correlation with NLR; the highest correlation was recorded between the number of TREC copies per 100,000 cells and NLR (Spearman’s ρ = −0.726, *p* = 1.0 × 10^−6^ coefficient of determination R^2^ = 0.527) (Supplementary Table S4).

## 4. Discussion

Our study examined the numbers of mature naive T and B cells in peripheral blood using TREC/KREC analysis in relation to ARDS development and mortality in COVID-19. The COVID-19 patients with ARDS are characterized by immune imbalance in which systemic inflammation and dysfunction of circulating T and B cells may be contributing to a more severe disease course [16]. The activated B cells (plasma cells) produce antibodies that neutralize extracellular viral particles and participate in the destruction of virus-infected cells, thus preventing virus binding and penetration into the cell. The cells infected with the virus can be recognized and destroyed by CD8+ cytotoxic T cells. CD4+ T cells have multiple effects, including stimulation of effective antiviral B and CD8+ T cell responses and regulation of innate and adaptive immunity, limiting immune-mediated organ damage [9]. Low levels of CD4+ and CD8+ T cells and B cells correlate with the severe course of COVID-19 and unfavorable outcome; the phenomenon of immune depletion mainly occurs with CD8+, to a lesser extent CD4+ T cells [17,18,19,20]. In COVID-19, there was a decreased ability to produce antiviral cytokines, particularly IFN-γ, and a shift of CD8+ T cells toward a terminally differentiated/senescent phenotype through reducing the number of naive (CD45RA+ CCR7+) and T central memory (Tcm, CD45RA- CCR7+) cells, whereas the frequency of terminally differentiated effector TEMRA (CD45RA+ CCR7-) cells and senescent (CD57+) CD8+ T cells was significantly higher compared to healthy controls. Among CD4+ T cells in these patients, naive, Tem, and TEMRA were mostly impaired, whereas Tcm were relatively intact [5]. Regulatory T cells (Treg), due to their suppressor properties, are able to limit inflammation and control immune homeostasis during viral infection. Treg numbers are significantly reduced in critically ill patients with COVID-19 compared to patients with mild disease [21]. Reduced Treg numbers in severe COVID-19 indicate inadequate regulation of proinflammatory immune responses, which can enhance hyperinflammation and tissue damage [22].

TREC and KREC levels were assessed per 100,000 cells and per leukocyte or lymphocyte counts per 1 μL of blood. Reduced TREC/KREC copies were registered for patients with ARDS compared to those without ARDS, and similar results were found for non-survivors. One of the reasons for low T and B cell levels, along with direct infection of T cells by SARS-CoV-2 and overproduction of inhibitory cytokines, may be the suppression of bone marrow hematopoiesis during “cytokine storm” [23].

During days 6 to 20 of treatment, higher NLR values were detected in patients with ARDS compared to the non-ARDS group. NLR is considered as an independent biomarker of disease severity and adverse clinical outcome [24,25,26]. Javanmard et al. showed that NLR > 6.5 was associated with 4-fold increase in the chance of severe COVID-19 and about a 1.8-fold increase of chance of fatal outcome [26]. TREC/KREC values were negatively correlated with NLR on the day of biomaterial collection; the highest correlation was recorded for TREC per 100,000 cells with a coefficient of determination R^2^ = 0.527. Consequently, we can suggest that mature naive T cells in the peripheral blood determine more than 50% of NLR values.

## 5. Conclusions

A limitation of our study is the sample size. The results obtained are preliminary, and further study of TREC/KREC in a larger sample will allow consideration of this parameter as a potential prognostic marker for the risk of development, severity, and outcome in the novel COVID-19 infection.

## Figures and Tables

**Table 1:** Baseline characteristics of hospitalized patients with COVID-19.

Parameter	Total Patients (*n* = 36)
Age, years	38.50 (31.00–44.00)
Male	21 (58.3%)
Hypertension	8 (22.22%)
Diabetes mellitus	3 (8.33%)
Body mass index, kg/m^2^	28.80 (23.90–40.90)
Missing, n (%)	14 (38.89%)
Time from onset of illness to hospitalization, days	6.50 (1.00–12.00)
Length of stay in hospital/ICU, days	15.50 (8.00–29.00)
SpO2, ≤93%	16 (44.44%)
Missing, n (%)	2 (5.56%)
Symptoms:	
Fever	22 (61.11%)
Cough	24 (66.67%)
Sore throat	4 (11.11%)
Chest tightness or chest pain	4 (11.11%)
Fatigue	31 (86.11%)
Chills	5 (13.89%)
Dyspnea	13 (36.11%)
Myalgia	4 (11.11%)
Vomiting or nausea	3 (8.33%)
Diarrhea	4 (11.11%)
Headache	6 (16.67%)
Loss of smell or taste	7 (19.44%)
CT score:	
0–1	16 (44.44%)
2	11 (30.56%)
3	6 (16.67%)
4	3 (8.33%)
ARDS:	10 (27.78%)
mild	2/10 (20.00%)
moderate	4/10 (40.00%)
severe	4/10 (40.00%)
Non-Survivors	4 (11.11%)

The data are presented as the median and interquartile range (10–90th percentiles) or as the total number and the percentage of patients with available data. ARDS—acute respiratory distress syndrome; CT—chest computed tomography score at the time of admission to hospital/ICU; SpO2—peripheral oxygen saturation. The ARDS categories are specified in accordance with the Berlin Definition [15].

**Table 2:** Comparison of TREC/KREC levels between ARDS and non-ARDS COVID-19 patients.

Variable	Non-ARDS (*n* = 26)	ARDS (*n* = 10)	*p*-Value	FDR Adjusted *p*-Value
TREC/100,000 cells	69.04 (15.43–230.60)	8.75 (0.00–259.56)	0.045	0.049
TREC/WBC in 1 µL of blood	4.40 (0.55–11.70)	0.76 (0.00–13.83)	0.049	0.049
TREC/lymphocytes in 1 µL of blood	0.09 (0.01–0.33)	0.01 (0.00–0.34)	0.026	0.039
KREC/100,000 cells	705.18 (86.64–1532.52)	160.76 (22.83–537.99)	0.002	0.006
KREC/WBC in 1 µL of blood	44.64 (5.50–127.93)	13.57 (2.10–29.74)	0.003	0.006
KREC/lymphocytes in 1 µL of blood	14.01 (1.99–39.48)	0.88 (0.23–39.48)	0.003	0.006

The data are presented as medians and interquartile range (10–90th percentiles). *p*-values for the differences between ARDS and non-ARDS patients were obtained from the Mann–Whitney U test.

## Data Availability

The data presented in this study are available on request from the corresponding author.

## Supplementary Materials

The following are available online at https://www.mdpi.com/article/10.3390/diagnostics11081486/s1, Table S1: Primer and probe sequences of TREC, KREC, and albumin. Table S2: Comparison of clinical characteristics and laboratory findings between ARDS and non-ARDS COVID-19 patients. Table S3: Comparison of TREC/KREC levels between survivor and non-survivor COVID-19 patients. Table S4: Correlation of TREC/KREC levels and NLR.
