# Supplementary material for: TREC/KREC Levels in Young COVID-19 Patients
Source: Diagnostics (Basel). 2021 Aug 16;11(8):1486. doi: 10.3390/diagnostics11081486 (PMC8392044; doi:10.3390/diagnostics11081486)
Supplement: Supplementary file 1 [file diagnostics-11-01486-s001.zip › diagnostics-1277735-supplementary.pdf]

Supplementary Materials

TREC/KREC Levels in Young COVID-19 Patients

Maryam B. Khadzhieva [1,2,3,*], Ekaterina V. Kalinina [2], Sergey S. Larin [2], Daria A. Sviridova [3], Alesya S. Gracheva [1,3], Julia V. Chursinova [4], Vadim A. Stepanov [4], Ivan V. Redkin [1], Lyudmila S. Avdeikina [5], Alexander G. Rumyantsev [2], Artem N. Kuzovlev [1] and Lyubov E. Salnikova [1,2,3]

[1] Federal Research and Clinical Center of Intensive Care Medicine and Rehabilitology, 107031 Moscow, Russia; palesa@yandex.ru (A.S.G.); redkin70@mail.ru (I.V.R.); artem_kuzovlev@fnkcrr.ru (A.N.K.); salnikovalyubov@gmail.com (L.E.S.)
[2] Dmitry Rogachev National Research Center of Pediatric Hematology, Oncology and Immunology, 117997 Moscow, Russia; ek.v.chernyshova@gmail.com (E.V.K.); sergei_larin@mail.ru (S.S.L.); alexrum47@mail.ru (A.G.R.)
[3] Vavilov Institute of General Genetics, Russian Academy of Sciences, 119991 Moscow, Russia; daria_sv11@mail.ru
[4] M.F. Vladimirsky Moscow Regional Research and Clinical Institute, 129110 Moscow, Russia; yu.chursinova@monikiweb.ru (J.V.C.); vedmak_@rambler.ru (V.A.S.)
[5] Moscow Clinical Center for Infectious Diseases "Voronovskoe", 142160 Moscow, Russia; saenko.doc@gmail.com
* Correspondence: mkhadzhieva@fnkcrr.ru; Tel.: +7-9636742099

Table S1. Primer and probe sequences of TREC, KREC, and albumin.

**Target**	**Primer or Probe**	**Sequence (5′ > 3′)**
TREC	Forward	CACATCCCTTTCAACCATGCT
	Reverse	TGCAGGTGCCTATGCATCA
	Probe	ACACCTCTGGTTTTTGTAAAGGTGCCCACT
KREC	Forward	TAAGCTTTCAGCGCCCATTA
	Reverse	CTCCAGGAGCCAGCTCTTAC
	Probe	CTGTCTCTTTCCCTTAGTGGCATTATTTG
ALB	Forward	TGAAACATACGTTCCCAAAGAGTTT
	Reverse	CTCTCCTTCTCAGAAAGTGTGCATAT
	Probe	TGAGATGCCTGCTGACTTGCCTTC A

Table S2. Comparison of clinical characteristics and laboratory findings between ARDS and non-ARDS COVID-19 patients.

**Parameter**	**non-ARDS (*n* = 26)**	**ARDS (*n* = 10)**	***p*-Value**	**FDR Adjusted *p*-Value**
Age, years	37.50 (32.00–44.00)	39.00 (25.50–44.50)	0.903	0.939
Male	15 (57.69%)	6 (60.00%)	0.931	0.939
Day of illness at the time of analysis	12.00 (8.00–20.00)	14.00 (7.50–28.00)	0.520	0.715
WBC, × 10^9/L	5.83 (2.85–10.30)	6.19 (4.15–14.84)	0.320	0.689
Lymphocyte count, × 10^9/L	1.73 (0.71–2.84)	0.86 (0.50–2.46)	**0.014**	0.077
Neutrophils, × 10^9/L	3.00 (1.42–8.56)	5.33 (2.90–14.37)	**0.049**	0.180
RBC, × 10^9/L	4.49 (4.02–5.34)	4.42 (3.44–5.37)	0.376	0.689
NLR	1.83 (0.70–10.31)	8.08 (1.15–29.94)	**0.002**	**0.022**
Platelets, × 10^9/L	280 (185–375)	270 (188–435)	0.939	0.939
IL-6R inhibitors (tocilizumab, olokizumab)	7 (26.92%)	4 (40.00%)	0.454	0.713
Corticosteroids (dexamethasone, prednisone, methylprednisolone)	10 (38.46%)	7 (70.00%)	0.139	0.382

The data are presented as median and interquartile range (10–90th percentiles) or n (%). *p*-values comparing ARDS and non-ARDS patients were obtained from Mann-Whitney U test and two-sided Fisher exact test. Significant differences ($p < 0.05$) are shown in bold. NLR—neutrophil-to-lymphocyte ratio; RBC—red blood cells; WBC—white blood cells.

Table S3. Comparison of TREC/KREC levels between survivor and non-survivor COVID-19 patients.

**Variable**	**Survivors (*n* = 32)**	**Non-Survivors (*n* = 4)**	***p*-Value**	**FDR Adjusted *p*-Value**
TREC/100000 cells	69.04 (4.05–247.31)	4.17 (0.94–9.71)*	0.008	0.016
TREC/WBC in 1 μL of blood	4.03 (0.23–12.87)	0.62 (0.14–1.10)*	0.018	0.018
TREC/lymphocytes in 1 μL of blood	0.08 (0.001–0.34)	0.003 (0.00–0.01)*	0.014	0.017
KREC/100000 cells	582.37 (79.01–1486.39)	66.61 (11.41–136.92)*	0.007	0.016
KREC/WBC in 1 μL of blood	32.99 (6.06–126.45)	4.58 (1.05–18.72)*	0.014	0.017
KREC/lymphocytes in 1 μL of blood	10.19 (0.83–38.10)	0.54 (0.11–0.71)*	0.003	0.016

The data are presented as median and interquartile range (10–90th percentiles). *p*-values for the differences between survivors and non-survivors were obtained from Mann-Whitney U test. *–median and (25–75th) percentiles.

Table S4. Correlation of TREC/KREC levels and NLR.

**Correlation Pairs**	***n***	**Spearman's ρ**	***p*-Value**
NLR & TREC/100000 cells	34	−0.726	0.000001
NLR & TREC/WBC in 1μL of blood	34	−0.595	0.000206
NLR & TREC/lymphocytes in 1μL of blood	34	−0.701	0.000004
NLR & KREC/100000 cells	34	−0.579	0.000330
NLR & KREC/WBC in 1μL of blood	34	−0.391	0.022390
NLR & KREC/lymphocytes in 1μL of blood	34	−0.691	0.000006
Missing	2		

n is the total number of patients with available data.
